# Demographic and Clinical Factors Associated with Bacterial or Nonbacterial Etiologies of Acute Undifferentiated Febrile Illness: Findings from a 3-Year Observational Study in Thailand, 2017–2020

**DOI:** 10.4269/ajtmh.23-0731

**Published:** 2024-07-16

**Authors:** Natalie R. Wodniak, Saithip Bhengsri, Beth Skaggs, Sumonmal Uttayamakul, Pongpun Sawatwong, Ornuma Sangwichian, Christopher J. Gregory, Nuttagarn Chuenchom, Pongpot Peanumlom, Supphachoke Khemla, Tanaphat Lertwitayakumjorn, Samkan Chaoprasert, Barameht Piralam, Tuwan Simmali, Chuwattana Chara, Emily Bloss, John R. MacArthur, James D. Heffelfinger

**Affiliations:** ^1^ Division of Global Health Protection, Thailand MOPH–US CDC Collaboration, Nonthaburi, Thailand;; ^2^ Bamrasnaradura Infectious Diseases Institute, Department of Disease Control, Ministry of Public Health, Nonthaburi, Thailand;; ^3^ Mae Sot General Hospital, Tak, Thailand;; ^4^ Nakhon Phanom Hospital, Nakhon Phanom, Thailand;; ^5^ Tak Provincial Health Office, Tak, Thailand;; ^6^ Nakhon Phanom Provincial Health Office, Nakhon Phanom, Thailand

## Abstract

Acute undifferentiated febrile illness (AUFI) is often undiagnosed in Thailand, resulting in delayed or ineffective treatment. We compared the demographic, exposure history, and clinical characteristics of AUFI patients with laboratory evidence of bacterial and nonbacterial pathogens. Patients aged 2–80 years presenting to 12 hospitals in Nakhon Phanom and Tak provinces were enrolled from April 2017 through May 2020. Interviews were conducted and blood, urine, and sputum were collected for culture as well as rapid diagnostic and molecular testing. A total of 1,263 patients tested positive for one or more bacterial, viral, or parasitic pathogens and were included in the analysis. Multivariable logistic regression was performed to compare factors associated with bacterial infections versus nonbacterial infections. Bacterial infections were more commonly identified in participants from Nakhon Phanom than Tak. Bacterial infections were independently associated with several factors including age ≥50 years (adjusted odds ratio [95% CI]): (4.18 [2.85–6.14]), contact with farm animals (1.82 [1.29–2.57]), antibiotic use within 72 hours of hospital presentation (2.37 [1.50–3.74]), jaundice (2.31 [1.15–4.63]), existing comorbidities (2.77 [1.93–3.96]), contact with febrile individuals (0.42 [0.31–0.57]), muscle pain (0.44 [0.31–0.64]), and rash (0.45 [0.29–0.70]). Bacterial infections were also associated with longer hospitalization (2.75 [2.08–3.64]) and lower odds of recovery at the time of discharge (0.14 [0.07–0.31]). Consideration of patient characteristics and signs/symptoms may help to inform targeted laboratory testing for suspected infectious etiologies. Understanding factors associated with bacterial and non-bacterial causes of AUFI may aid diagnosis and judicious use of antibiotics in resource-limited settings.

## INTRODUCTION

Acute febrile illness (AFI) is one of the most common reasons for hospitalization and emergency department visits globally.^1^^,^^2^ Febrile illnesses can have a wide range of etiologies, including bacterial, parasitic, and viral infections, as well as systemic conditions.^3^^–^^5^ The ability to correctly diagnose the cause of fever has critical implications for both patient management and public health because accurate diagnosis can ensure that proper treatment is administered in a timely fashion and can limit further spread of infectious diseases.^1^^,^^2^^,^^6^^,^^7^ However, it is common for the etiologies of AFI in hospitalized patients to remain unidentified or to be misdiagnosed, especially in low- and middle-income countries and settings with limited laboratory capacity, in part due to the wide range of causes of febrile illnesses and similarity of symptoms.^2^^,^^4^^,^^7^

Inaccurate diagnosis can lead to the overuse of antimicrobials, contributing to the growing issue of antimicrobial resistance (AMR) in Thailand and Southeast Asia.^8^^,^^9^ In Thailand, over-prescription of antibiotics in health facilities is common, and misuse of antibiotics also occurs due to the availability of antimicrobials over-the-counter in pharmacies.^10^^–^^14^ Additionally, health literacy about antibiotics and AMR is generally low among adults in Thailand,^15^^,^^16^ and rates of inappropriate antibiotic use are high.^10^^,^^14^

Acute febrile illness can present without localized manifestations such as respiratory or diarrheal signs and symptoms, in which case it is referred to as acute undifferentiated febrile illness (AUFI), which can be particularly difficult to diagnose.^1^^,^^17^ In Thailand and Southeast Asia, the numerous infectious etiologies of AUFI include, but are not limited to, dengue virus, chikungunya virus, Zika virus, *Salmonella enterica*, *Burkholderia pseudomallei*, *Orientia tsutsugamushi* (the cause of scrub typhus), pathogenic *Leptospira* species, and *Rickettsia* species.^2^^,^^5^^,^^18^^–^^23^ Due to the high prevalence of dengue fever in the region, AUFI is frequently misdiagnosed as dengue, which can impede the proper management of illness and delay potentially lifesaving treatment.^19^^,^^24^

**Figure 1.**
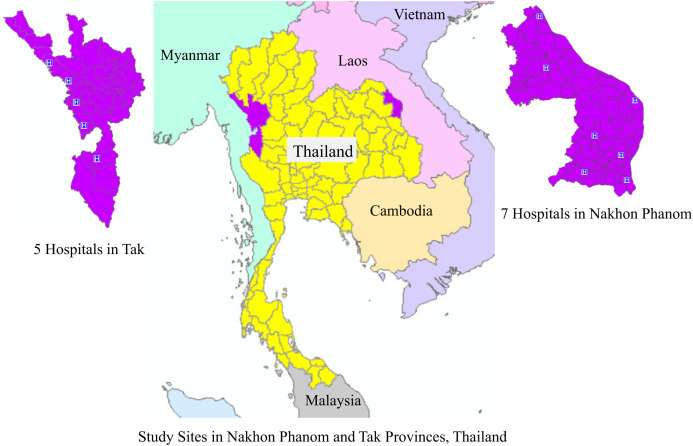
Study sites in Nakhon Phanom and Take provinces, Thailand.

Several previous studies have investigated clinical and hematological indicators of bacterial versus nonbacterial infections but few have assessed patients’ risk factors and exposure histories or focused specifically on AUFI.^6^^,^^25^^–^^29^ The majority of previous studies in Thailand have analyzed clinical and laboratory factors associated with specific disease diagnoses, such as comparing dengue to chikungunya, scrub typhus, or typhoid.^30^^–^^34^ To our knowledge, no studies in Thailand have compared clinical predictors and exposure histories among patients diagnosed with bacterial and nonbacterial febrile illnesses. An improved ability to determine whether an undifferentiated illness is bacterial or nonbacterial in origin can hasten proper management, which may have three primary results: 1) improve patient outcomes, 2) reduce the chance of further transmission of infectious disease in the surrounding community, and 3) reduce the unnecessary use of antimicrobial treatments. This analysis aims to assess sociodemographic, clinical, and risk factors associated with bacterial and nonbacterial infections in AUFI patients in two border provinces of Thailand (Nakhon Phanom, borders Laos; Tak, borders Myanmar, Figure 1).

Results from this analysis may help to increase local knowledge of the characteristics of patients presenting to hospitals with bacterial and nonbacterial causes of AUFI. Findings may aid in the proper screening and treatment of fever in Nakhon Phanom and Tak based on demographic, exposure, and clinical risk factors.

## MATERIALS AND METHODS

### Project design and study population.

From April 2017 to May 2020, a prospective observational study was conducted among patients aged 2–80 years hospitalized with AFI in 12 healthcare facilities in Nakhon Phanom and Tak (five hospitals along the borders) provinces (Supplemental Table 1). Acute febrile illness was defined as fever (temperature ≥38°C upon admission) or history of fever (subjective or measured) with onset ≤7 days before admission. Patients were excluded if they were returning to the hospital for continuation of treatment of fever within 30 days, had been admitted to any hospital in the previous 14 days, or could not read or understand Thai, Lao, Burmese, or Karen languages. Patients with AFI who had evidence of fewer than two clinical respiratory signs or symptoms (defined as sore throat, rhinitis, cough, difficulty breathing, and sputum production) and no evidence of diarrheal disease (defined as a clinical diagnosis of diarrhea or three or more loose or watery stools within the previous 24 hours reported by the patient or guardian) were categorized as having AUFI. This analysis was restricted to patients with AUFI.

#### Data and specimen collection.

Written informed consent was obtained from all patients aged ≥18 years and from guardians of patients <18 years of age, and assent was obtained from children aged 7–17 years. Project staff conducted interviews and reviewed medical records to collect information on patient demographics, clinical signs and symptoms, exposure history, underlying comorbidities, laboratory results, and clinical course during hospital stays. All AUFI patients had blood and urine samples collected within 24 hours of hospital admission, and participants ≥18 years with any respiratory symptoms also had sputum specimens collected, if possible.

### Laboratory diagnostics.

Blood samples were tested for bacterial pathogens using an automated blood culture system (BD BACTEC^TM^ FX, BD Franklin Lakes, NJ) and automated identification and susceptibility testing system (BD Phoenix^TM^, BD). Sputum and urine were cultured for bacterial pathogens using conventional methods. Rapid diagnostic tests (RDT) were used to test urine samples for *Streptococcus pneumoniae* (Binax NOW^®^ *S. pneumoniae* Antigen Card, Abbott, Chicago, IL), and blood samples for dengue (SD Bioline Dengue Duo, Abbott), and malaria (Humasis Malaria Pf/Pan Antigen Test, Humasis, York, United Kingdom). In addition, singleplex real-time polymerase chain reaction assays (RT-qPCR, 7500 Real-Time PCR System, Thermo Fisher Scientific, Waltham, MA) were used to detect bacterial and viral pathogens known to cause febrile illness in Thailand as well as specific dengue serotypes. Supplemental Table 2 details the PCR targets for each pathogen.

### Definitions.

Only individuals with positive laboratory results were considered for this analysis. The following definitions were used:

- Dengue virus infections—NS1 antigen positive and/or IgM positive by RDT, and/or dengue serotype positive by RT-qPCR
- Malaria infections—RDT positive for *Plasmodium falciparum*/pan *Plasmodium*
- Chikungunya virus, Zika virus, pathogenic *Leptospira* spp., *O. tsutsugamushi*, *Rickettsia rickettsii*, and pan-*Rickettsia* infections—RT-qPCR or qPCR positive
- *Escherichia coli*, *Klebsiella pneumoniae*, *Streptococcus pyogenes*, *Streptococcus agalactiae*, *Staphylococcus aureus*, *Acinetobacter baumannii*, *Streptococcus suis*, *Haemophilus influenzae*, *Pseudomonas aeruginosa* infections: hemoculture, urine or sputum culture, and/or qPCR positive
- *S. pneumoniae* infections—BINAX RDT positive, hemoculture, urine or sputum culture positive
- *B. pseudomallei* infections—hemoculture, urine or sputum culture, and/or qPCR positive
- Japanese encephalitis virus infection—IgM positive by ELISA
- Other *Streptococcus* spp. infection—hemoculture, urine or sputum culture positive
- Other bacterial infection—any other bacterial pathogens detected from hemoculture, urine culture, sputum culture, and/or qPCR

For this analysis, patients with laboratory-confirmed evidence of bacterial or nonbacterial infections:

1. Bacterial infections included laboratory-positive results for leptospirosis (*Leptospira* spp.), scrub typhus (*O. tsutsugamushi*), rickettsiosis (*Rickettsia* spp.), melioidosis (*B. pseudomallei*), *E. coli*, *K. pneumoniae*, *S. pyogenes*, *S. agalactiae*, *S. aureus*, *A. baumannii*, *S. suis*, *H. influenzae*, *P. aeruginosa*, other *Streptococcus* spp., and other bacterial detections.
  a. Patients with laboratory-confirmed multiple bacterial pathogens, in the absence of nonbacterial pathogens, were classified as having bacterial infection.
2. Nonbacterial infections included laboratory-positive results for dengue virus, chikungunya virus, malaria, Zika virus, and Japanese encephalitis virus.
  a. Patients with laboratory-confirmed multiple nonbacterial pathogens, in the absence of bacterial pathogens, were classified as having nonbacterial infection.
3. Patients with laboratory-confirmed both bacterial and nonbacterial pathogens were excluded from the analysis.

## STATISTICAL ANALYSES

Descriptive analyses were performed for the distribution of pathogens detected and the demographic characteristics of patients with bacterial or nonbacterial infections. Median and interquartile range (IQR) were applied for continuous variables with non-normal distribution. Chi-square tests were performed to compare the proportion of laboratory-confirmed evidence for pathogen between Nakhon Phanom and Tak provinces. Statistical significance was set at *P* ≤0.05.

Categorical variables that were similar in nature (e.g., contact with cows/contact with pigs/contact with goats/contact with sheep; contact with febrile household member/contact with febrile coworker/contact with febrile neighbor) and showing the same direction of association in bivariate models were combined to reduce the number of predictors included in multivariable models. Comorbidities identified from medical records (including diabetes, hypertension, heart disease, asthma, chronic obstructive pulmonary disease, cancer, HIV, immunodeficiency, history of tuberculosis, active tuberculosis, liver disease, thyroid disease, thalassemia, anemia, chronic renal disease, and other chronic disease) were combined into one variable specifying the presence of any comorbidities.

Simple logistic regressions were conducted to assess the predictive effects of demographic variables, exposure history, symptoms, antibiotic use, and comorbidities on bacterial versus nonbacterial infections in both provinces combined. All variables with bivariate significance of *P* ≤0.2 were considered for inclusion into a multivariable model. Multicollinearity among predictor variables was assessed through the variance inflation factor (VIF) with a cutoff value of ≤5.^35^ A multivariable logistic regression model was fit using backward selection, retaining variables with *P* ≤0.05. Odds ratios (ORs), adjusted ORs (aORs), and their corresponding 95% CIs were calculated for variables included in the multivariable model.

To assess whether findings may have been driven by commonly detected pathogens, bivariate and multivariable logistic regression analyses were conducted for the most detected bacterial and nonbacterial pathogens. We compared characteristics of individuals with any evidence of each common pathogen (regardless of whether multiple bacterial or nonbacterial pathogens were identified) to those with no evidence to the pathogen of interest. Results were compared with the primary multivariable analysis of bacterial versus nonbacterial infections.

Clinical outcomes and severity of illness were assessed using simple logistic regression to examine associations between days of hospitalization, intubation/mechanical ventilation, and discharge status among patients with bacterial versus nonbacterial infections. Associations were further explored in multivariable models including a priori adjustments for age and the presence of comorbidities.

A sensitivity analysis was performed to examine differences between Nakhon Phanom and Tak: bivariate and multivariable logistic regressions were performed for each province separately to assess whether associations appreciably differed by province. An additional sensitivity analysis was conducted by performing multivariable regression stratified by age group.

All data were analyzed using SAS Version 9.4 (Cary, NC).

## RESULTS

**Figure 2.**
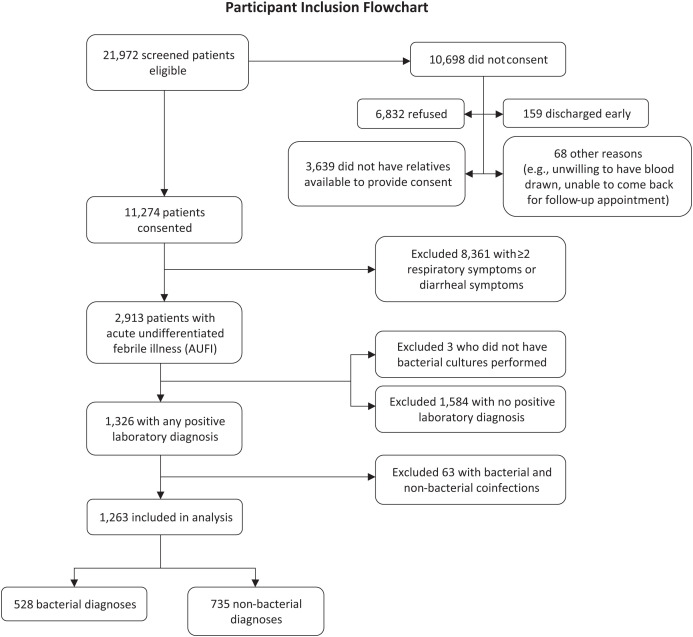
Participant inclusion chart.

During the study period, 21,972 patients with AFI were eligible to participate in the study, and 11,274 (51.3%) consented. Among patients who did not consent, 31% (6,832/21,972) refused to participate, and we were not allowed to enroll children who did not have parents available to provide consent, 16% (3,639/21,972) (Figure 2). Overall, 2,913 (25.8%) of consenting patients presented with AUFI (*N* = 1,881 in Nakhon Phanom and *N* = 1,032 in Tak), and 1,326 (45.5%) of these patients had specimens with laboratory-confirmed evidence of one or more pathogens. Among individuals with bacterial or nonbacterial infections, 63 (4.8%) tested positive for both bacterial and nonbacterial pathogens and were excluded, resulting in 1,263 individuals included in the analysis (Figure 2).

### Participant characteristics.

Demographic and clinical characteristics of AUFI patients are shown in Table 1. The median age of AUFI patients was 42 years (IQR: 20–60). Of 1,263 patients, the majority (93.9%) were of Thai nationality, and farming was the most common occupation (31.8%). Patients had an average of 2.1 days of fever (SD: 1.7) before presenting to the hospital, and the most common symptoms in addition to fever were fatigue (86.4%), chills (77.0%), and muscle pain (71.6%). A total of 31.8% of patients reported one or more underlying comorbidities, with hypertension (15.4%), diabetes (13.3%), and chronic renal disease (8.6%) being the most frequently reported among participants.

**Table 1:** Demographic and clinical characteristics of patients with acute undifferentiated fever, Nakhon Phanom and Tak provinces, Thailand, April 2017–May 2020

Characteristics	Overall (*N* = 1,263)		Bacterial Infections (*n* = 528)		Non-bacterial Infections (*n* = 735)		Bacterial vs. Nonbacterial Infections	
	*n*	%	*n*	%	*n*	%	OR (95% CI)	*P*-Value
Province of Hospital								
Nakhon Phanom	720	57.0	386	73.1	334	45.4	**3.26 (2.57–4.15)**	**<0.0001**
Tak	543	43.0	142	26.9	401	54.6	–	–
Sex								
Male	620	49.1	261	49.4	359	48.8	0.98 (0.78–1.22)	0.84
Female	643	50.9	267	50.6	376	51.2	–	–
Age, years								**<0.0001**
2–17	283	22.4	49	9.3	234	31.8	**0.54 (0.38–0.78)**	**<0.01**
18–49	550	43.6	153	29.0	397	54.0	Ref.	Ref.
≥50	430	34.1	326	61.7	104	14.2	**8.13 (6.09–10.86)**	**<0.0001**
Nationality								
Thai	1187	93.9	501	94.9	686	93.3	1.33 (0.82–2.15)	0.25
Other	76	6.0	27	5.1	49	6.7	–	–
Employment Status								**<0.0001**
Employed*	791	62.6	358	67.8	433	58.9	Ref.	Ref.
Student/Preschool Student	311	24.6	52	9.9	259	35.2	**0.24 (0.18–0.34)**	**<0.0001**
Unemployed	161	12.8	118	22.4	43	5.9	**3.32 (2.28–4.84)**	**<0.0001**
Year of Study								**<0.001**
Year 1 (April 2017–March 2018)	382	30.3	183	34.7	199	27.1	**2.10 (1.60–2.76)**	**<0.001**
Year 2 (April 2018–March 2019)	345	27.3	182	34.5	163	22.2	**2.56 (1.96–3.38)**	**<0.001**
Year 3 (April 2019–May 2020)	536	42.4	163	30.9	373	50.8	Ref.	Ref.
Hospital Type								
Provincial	512	40.5	212	40.2	300	40.8	–	–
District	751	59.5	316	59.9	435	59.2	1.03 (0.82–1.29)	0.81
Contact with Febrile Household Member, Coworker, or Neighbor	572	45.3	159	30.1	413	56.2	**0.34 (0.27–0.43)**	**<0.0001**
Contact with Farm Animal (Cow, Pig, Goat, or Sheep)	348	27.6	169	32.0	179	24.4	**1.46 (1.14–1.88)**	**<0.01**
Contact with Chicken or Duck	656	51.9	323	61.2	333	45.3	**1.90 (1.52–2.39)**	**<0.0001**
Contact with Cats or Dogs	800	63.3	341	64.6	459	62.5	1.10 (0.87–1.38)	0.44
Contact with Rodents	592	46.9	288	54.6	304	41.4	**1.70 (1.36–2.13)**	**<0.0001**
Contact with Stray Animal^†^	118	9.3	63	11.9	55	7.5	**1.68 (1.15–2.45)**	**<0.01**
Bitten by Mosquitoes or Other Insects	1244	98.5	516	97.7	728	99.1	0.41 (0.16–1.06)	0.07
Contact with Flood Water, Mud, Ponds, or Rivers	435	34.4	188	35.6	247	33.6	1.09 (0.86–1.38)	0.46
Cut or Scraped Self	179	14.2	68	12.9	111	15.1	0.83 (0.60–1.15)	0.26
Cut Down Trees, Bushes, Gathered Wood, and/or Cleared Land	336	26.6	157	29.7	179	24.4	**1.31 (1.02–1.69)**	**0.03**
Ate raw or Undercooked Fish or Pork	319	25.3	153	29.0	166	22.6	**1.40 (1.08–1.81)**	**0.01**
Visited Forest	386	30.6	158	29.9	228	31.0	0.95 (0.75–1.21)	0.68
Visited or Worked with Rubber Trees	145	11.5	65	12.3	80	10.9	1.15 (0.81–1.63)	0.43
Walked Outside with no Shoes	413	32.7	188	35.6	225	30.6	1.25 (0.99–1.59)	0.06
Antibiotics Taken within 72 hours before Hospital Presentation								
Yes^‡^	152	12.0	79	15.0	73	9.9	**1.58 (1.13–2.22)**	**<0.01**
No	1073	85.0	436	82.6	637	86.7	Ref.	Ref.
Unknown	7	0.6	5	1.0	2	0.3	–	–
Not Asked	31	2.5	8	1.5	23	3.1	–	–
Days of Fever Before Hospital Admission								
0–1	425	33.7	214	40.5	211	28.7	**1.50 (1.15–1.95)**	**<0.0001**
2–3	483	38.2	195	36.9	288	39.2	Ref.	Ref.
4–7	355	28.1	119	22.5	236	32.1	**0.75 (0.56–0.99)**	**<0.01**
Symptoms								
Cough	141	11.2	63	11.9	78	10.6	1.14 (0.80–1.62)	0.46
Rhinitis	37	2.9	14	2.7	23	3.1	0.84 (0.43–1.65)	0.62
Sore Throat	64	5.1	25	4.7	39	5.3	0.89 (0.53–1.49)	0.65
Shortness of Breath/Difficulty Breathing	137	10.9	76	14.4	61	8.3	**1.89 (1.30–2.66)**	**<0.01**
Nausea or Vomiting	702	55.6	253	47.9	449	61.1	**0.59 (0.47–0.74)**	**<0.0001**
Yellow Eyes or Skin	66	5.2	45	8.5	21	2.9	**3.17 (1.86–5.39)**	**<0.0001**
Headache	951	75.3	358	67.8	593	80.7	**0.50 (0.39–0.65)**	**<0.0001**
Blood in Urine, Stool, or Vomit	68	5.4	30	5.7	38	5.2	1.11 (0.68–1.81)	0.69
Muscle Pain	904	71.6	360	68.2	544	74.0	**0.75 (0.59–0.96)**	**0.02**
Chest Pain	190	15.0	93	17.6	97	13.2	**1.41 (1.03–1.92)**	**0.03**
Bone or Joint Pain	550	43.6	227	43.0	323	44.0	0.96 (0.77–1.21)	0.74
No Appetite	947	75.0	378	71.6	569	77.4	**0.74 (0.57–0.95)**	**0.02**
Tiredness, No Energy	1091	86.4	460	87.1	631	85.9	1.12 (0.80–1.55)	0.52
Seizures	30	2.4	14	2.7	16	2.2	1.22 (0.59–2.53)	0.59
Chills	972	77.0	421	79.7	551	75.0	1.31 (1.00–1.72)	0.05
Pale or Cold Skin	429	34.0	210	39.8	219	29.8	**1.56 (1.23–1.97)**	**<0.01**
Rash	239	18.9	38	7.2	201	27.4	**0.21 (0.14–0.30)**	**<0.0001**
Bruises	27	2.1	11	2.1	16	2.2	0.96 (0.44–2.08)	0.91
Any Existing Comorbidities^§^	402	31.8	295	55.9	107	14.6	**7.43 (5.69–9.71)**	**<0.0001**
Current Smoking	184	14.6	95	18.0	89	12.1	**1.59 (1.16–2.18)**	**<0.01**
Current Alcohol Consumption	250	19.8	107	20.3	143	19.5	1.05 (0.80–1.39)	0.72

– = not included in the analysis; OR = odds ratio; Ref. = reference. Bold value indicates *P* <0.05.

* Used occupations included farmer, healthcare personnel, government/office worker, merchant, monk, housekeeper, and laborer.

^†^ Streets or homeless animal.

^‡^ Of 152 patients, 27 (17.8%) patients aged 2–17 years, 73 (48.0%) aged 18–49 years, and 52 (34.2%) aged ≥50 years.

^§^ Comorbidities included diabetes, hypertension, heart disease, asthma, chronic obstructive pulmonary disease, cancer, HIV, immunodeficiency, history of tuberculosis, active tuberculosis, liver disease, thyroid disease, thalassemia, anemia, chronic renal disease, and other chronic disease.

### Laboratory results.

Among 1,263 AUFI patients, 528 (41.8%) tested positive for one or more bacterial pathogens, and 735 (58.2%) tested positive for one or more nonbacterial pathogens. In both provinces, *E. coli* was the most common bacterial pathogen detected, identified in 160 (12.7%) patients, and dengue virus was the most common nonbacterial pathogen detected, identified in 563 (44.6%) patients (Table 2). Thirty-six patients (2.9%) tested positive for multiple bacterial pathogens, and 115 patients (9.1%) tested positive for multiple nonbacterial pathogens (Supplemental Table 3).

**Table 2:** Distribution of laboratory-confirmed diagnoses in Nakhon Phanom and Tak provinces, Thailand, April 2017–May 2020

Pathogen	Nakhon Phanom (*n* = 720)	Tak (*n* = 543)	*P*-Value	Total (*N* = 1,263)
	*n* (%)*	*n* (%)*		*n* (%)*
Bacterial Detections^†^ (*N* = 528; Nakhon Phanom: *n* = 386, Tak: *n* = 142)				
*Orientia tsutsugamushi*	4 (0.6)	32 (5.9)	<0.0001	36 (2.9)
Pathogenic *Leptospira* Species	44 (6.1)	27 (5.0)	0.38	71 (5.6)
*B. pseudomallei*	26 (3.6)	0 (0)	<0.0001	26 (2.1)
*Rickettsia* spp.	59 (8.2)	10 (1.8)	<0.0001	69 (5.5)
*Escherichia coli*	119 (16.5)	41 (7.6)	<0.0001	160 (12.7)
*Krebseilla pneumoniae*	27 (3.8)	3 (0.6)	<0.01	30 (2.4)
*Streptococcus agalactiae*	13 (1.8)	1 (0.2)	<0.01	14 (1.1)
*Streptococcus pneumoniae*	9 (1.3)	9 (1.7)	0.55	18 (1.4)
*Streptococcus pyogenes*	3 (0.4)	1 (0.2)	0.64^‡^	4 (0.32)
*Streptococcus aureus*	20 (2.8)	7 (1.3)	0.07	27 (2.1)
*Acinetobacter baumannii*	4 (0.6)	0 (0)	0.14^‡^	4 (0.3)
*Streptococcus suis*	3 (0.4)	0 (0)	0.26^‡^	3 (0.2)
*Haemophilus influenza*	1 (0.1)	0 (0)	1.00^‡^	1 (0.1)
*Psedomonas aeruginosa*	2 (0.3)	0 (0)	0.51^‡^	2 (0.2)
Other *Streptococcus* Species	12 (1.7)	3 (0.6)	0.07	15 (1.2)
Other Bacteria (Culture Signaled Positive)	70 (9.7)	16 (3.0)	<0.0001	86 (6.8)
Multiple Bacterial Detections	29 (4.0)	7 (1.3)	<0.01	36 (2.9)
Nonbacterial Detections (*N* = 735; Nakhon Phanom: *n* = 334, Tak: *n* = 401)				
Dengue Virus	288 (40.0)	275 (50.6)	<0.01	563 (44.6)
Chikungunya Virus	28 (3.9)	96 (17.7)	<0.0001	124 (9.8)
Malaria (*Plamodium falciparum*/Pan *Plasmodium*)	0 (0)	30 (2.4)	<0.0001	26 (2.1)
Zika Virus	1 (0.1)	2 (0.4)	0.58^‡^	3 (0.2)
Japanese Encephalitis Virus^§^	66 (9.2)	65 (12.0)	0.11	131 (10.4)
Multiple Nonbacterial Detections	49 (6.8)	66 (12.2)	<0.01	115 (9.1)

* Percentages do not add up to 100% because of cases with more than one bacterial or nonbacterial detection.

^†^ *R. rikettsii* were not detected.

^‡^ Calculated using Fisher’s exact test.

^§^ Cross-reactivity between Japanese encephalitis and dengue IgM was not accounted for because both were considered nonbacterial detections.

Demographic characteristics and exposure histories differed for patients with laboratory-confirmed evidence for the three most common pathogens: dengue virus, *E. coli*, and chikungunya virus. Notably, dengue virus was found in 70.0% (198/283) of children aged 2–17 years, and *E. coli* was found in 28.6% (123/430) of individuals aged ≥50. Chikungunya virus comprised 17.7% (96/654) of all laboratory-confirmed nonbacterial infection in Tak province, although it was only found during year 3 of the study (Supplemental Table 4).

The proportion of bacterial infections was higher in Nakhon Phanom (53.6%, 386/720) than in Tak (26.2%, 142/543; *P <*0.0001). *Burkholderia pseudomallei*, *A. baumannii*, *S. suis*, *H. influenzae*, and *P. aeruginosa* were only found in Nakhon Phanom, and malaria was only found in Tak. Of patients with bacterial infections, *B. pseudomallei*, *Rickettsia* spp., *E. coli*, *K. pneumoniae*, *S. agalactiae*, and other bacteria were significantly more common in Nakhon Phanom (*P <*0.05), and *O. tsutsugamushi* was more common in Tak (*P <*0.0001). Of patients with nonbacterial detections, dengue virus, chikungunya virus, and malaria were more common in Tak (*P <*0.01; Table 2).

### Bivariate analysis.

Factors associated with infections type are shown in Table 1. Age ≥50 years was associated with bacterial infections (*P* <0.01), and the youngest age group (2–17 years) was associated with the nonbacterial infections (*P* <0.0001) compared with the 18- to 49-year age group. Compared with individuals who were employed, students and preschoolers had greater odds of nonbacterial infections, and unemployed individuals had greater odds of bacterial infection (*P* <0.0001). Bacterial and nonbacterial infections did not significantly differ by sex, nationality, or type of hospital (provincial or district).

Bacterial infection was associated with exposure to farm animals, poultry, rodents, and stray animals, as well as a recent history of cutting down trees and eating raw or undercooked fish or pork (*P <*0.05). Contact with febrile household members, coworkers, or neighbors was associated with nonbacterial infection (*P <*0.0001).

Bacterial infections were more common among individuals who had taken antibiotics in the 72 hours before presentation to the hospital (*P <*0.01). Shortness of breath, jaundice, chest pain, chills, pallor, fewer days of fever before hospital admission, presence of comorbidities, and smoking were associated with bacterial infection, whereas nausea and/or vomiting, headache, muscle pain, decreased appetite, and rash were associated with nonbacterial infection (*P <*0.05).

### Multivariable analysis.

Results from the multivariable analysis are shown in Table 3. The VIF for all variables was <5, and multicollinearity was determined to be unlikely to impact the multivariable model. Patients in Nakhon Phanom province had greater odds of bacterial infection than patients in Tak (aOR: 2.82, 95% CI: 2.02–3.93). Bacterial infection was independently associated with age ≥50 years (aOR: 4.18, 95% CI: 2.85–6.14), unemployment (aOR: 1.68, 95% CI: 1.01–2.79), study years 1 (aOR: 2.92, 95% CI: 2.01–4.24) and 2 (aOR: 3.30, 95% CI: 2.25–4.82), contact with farm animals (aOR: 1.82, 95% CI: 1.29–2.57), antibiotic use within 72 hours prior to hospital presentation (aOR: 2.37, 95% CI: 1.50–3.74), jaundice (aOR: 2.31, 95% CI: 1.15–4.46), chest pain (aOR: 1.79, 95% CI: 1.18–2.73), pallor (aOR: 1.70, 95% CI: 1.23–2.35), and existing comorbidities (aOR: 2.77, 95% CI: 1.93–3.96). Contact with febrile individuals (aOR: 0.42, 95% CI: 0.31–0.57), nausea and/or vomiting (aOR: 0.73, 95% CI: 0.54–1.00), muscle pain (aOR: 0.44, 95% CI: 0.31–0.64), and rash (aOR: 0.45, 95% CI: 0.29–0.70) were associated with lower odds of bacterial infection.

**Table 3:** Demographic, exposure, and clinical predictors for bacterial versus nonbacterial infections, Nakhon Phanom and Tak provinces, Thailand, April 2017–May 2020

Predictor	aOR* (95% CI)
Province of Hospital	
Nakhon Phanom	**2.82 (2.02–3.93)**
Tak	Ref.
Age, years	
2–17	1.16 (0.48–2.77)
18–49	Ref.
≥50	**4.18 (2.85–6.14)**
Employment Status	
Employed	Ref.
Student/Preschool	0.50 (0.21–1.16)
Unemployed	**1.68 (1.01–2.79)**
Year of Study	
Year 1 (April 2017—March 2018)	**2.92 (2.01–4.24)**
Year 2 (April 2018–March 2019)	**3.30 (2.25–4.82)**
Year 3 (April 2019–May 2020)	Ref.
Contact with Febrile Household Member, Neighbor, or Coworker	**0.42 (0.31–0.57)**
Contact with Cow, Pig, Goat, or Sheep	**1.82 (1.29–2.57)**
Antibiotics Taken within 72 hours before Hospital Presentation	
Yes	**2.37 (1.50–3.74)**
No	Ref.
Yellow Eyes or Skin	**2.31 (1.15–4.63)**
Nausea and/or Vomiting	**0.73 (0.54–1.00)**
Muscle Pain	**0.44 (0.31–0.64)**
Chest Pain	**1.79 (1.18–2.73)**
Pallor	**1.70 (1.23–2.35)**
Rash	**0.45 (0.29–0.70)**
Any Existing Comorbidities^†^	**2.77 (1.93–3.96)**

aOR = adjusted odds ratio; Ref. = reference.

* Adjusted for all other variables included in the model.

^†^ Comorbidities include diabetes, hypertension, heart disease, asthma, COPD, cancer, HIV, immunodeficiency, history of tuberculosis, active tuberculosis, liver disease, thyroid disease, thalassemia, anemia, chronic renal disease, and other chronic disease.

Multivariable results for factors associated with *E. coli* and dengue virus infections are shown in Table 4. Similar to the overall multivariable analysis, *E. coli* infection was independently associated with age ≥50 years (aOR: 2.72, 95% CI: 1.68–4.41), pallor (aOR: 1.54, 95% CI: 1.03–2.32), and existing comorbidities (aOR: 2.43, 95% CI: 1.56–3.78); lower odds of *E. coli* infection were observed among those with contact with febrile individuals (aOR: 0.53, 95% CI: 0.35–0.82). Dengue virus infection was independently associated with contact with febrile individuals (aOR: 1.74, 95% CI: 1.28–2.40), nausea and/or vomiting (aOR: 1.70, 95% CI: 1.23–2.34), hematocrit >40 mg% (aOR: 2.21, 95%CI: 1.56–3.12), white blood cell count <4,000/mm^3^ (aOR: 6.48, 95% CI: 4.45–9.43), and platelet count <100,000/mm^3^ (aOR: 2.66, 95% CI: 1.84–3.84). Age ≥50 years (aOR: 0.33, 95% CI: 0.22–0.50), antibiotic use within 72 hours before hospital admission (aOR: 0.65, 95% CI: 0.41–1.05), chest pain (aOR: 0.48, 95% CI: 0.31–0.75), pallor (aOR: 0.66, 95% CI: 0.48–0.92), and existing comorbidities (aOR: 0.47, 95% CI: 0.31–0.69) were associated with lower odds of dengue virus infection. Additional associations between the infection of each pathogen and variables not included in the bacterial versus nonbacterial multivariable analysis are reported in Table 4 (see Supplemental Table 4 for bivariate results).

**Table 4:** Factors associated with the most common bacterial (*E. coli*) and nonbacterial (dengue virus) infections, Nakhon Phanom and Tak provinces, Thailand, April 2017–May 2020

Characteristics	Dengue Virus (*N* = 563)	*E. coli* (*N* = 160)
	aOR* (95% CI)	aOR* (95% CI)
Female Sex	–	**2.82 (1.82–4.37)**
Age, years		
2–17	**2.08 (1.38–3.12)**	**0.06 (0.01–0.48)**
18–49	Ref.	Ref.
≥50	**0.33 (0.22–0.50)**	**2.72 (1.68–4.41)**
Year of Study		
Year 1 (April 2017–March 2018)	**1.52 (1.04–2.22)**	–
Year 2 (April 2018–March 2019)	0.94 (0.64–1.40)	–
Year 3 (April 2019–May 2020)	Ref.	–
Contact with Febrile Household Member, Coworker, or Neighbor	**1.74 (1.28–2.40)**	**0.53 (0.35–0.82)**
Contact with Cats or Dogs	–	**1.68 (1.10–2.57)**
Antibiotics Taken within 72 hours before Hospital Presentation^†^		
Yes	**0.65 (0.41–1.05)**	–
No	Ref.	–
Days of Fever before Hospital Admission		
0–1	**1.15 (0.77–1.72)**	1.01 (0.65–1.56)
2–3	Ref.	Ref.
4–7	**1.38 (0.95–2.00)**	**0.55 (0.30–1.02)**
Nausea and/or Vomiting	**1.70 (1.23–2.34)**	–
Headache	**1.51 (1.03–2.21)**	**0.53 (0.35–0.83)**
Chest Pain	**0.48 (0.31–0.75)**	–
Bone or Joint Pain	**0.66 (0.47–0.92)**	–
No Appetite	–	**0.57 (0.37–0.88)**
Chills	–	**1.76 (1.02–3.02)**
Pale or Cold Skin	**0.66 (0.48–0.92)**	**1.54 (1.03–2.32)**
Rash	1.40 (0.95–2.07)	–
Any Existing Comorbidities^‡^	**0.47 (0.31–0.69)**	**2.43 (1.56–3.78)**
Hematocrit >40 mg%	**2.21 (1.56–3.12)**	**0.59 (0.32–1.09)**
White Blood Cell <4,000/mm^3^	**6.48 (4.45–9.43)**	**0.68 (0.36–1.29)**
Platelet <100,000/mm^3^	**2.66 (1.84–3.84)**	**0.47 (0.26–0.86)**

– = not included in multivariable model; aOR = adjusted odds ratio; *E. coli* = *Escherichia coli*; OR = odds ratio; Ref. = reference.

* Adjusted for all other variables included in the model.

^†^ Excluded unknown.

^‡^ Comorbidities included diabetes, hypertension, heart disease, asthma, chronic obstructive pulmonary disease, cancer, HIV, immunodeficiency, history of tuberculosis, active tuberculosis, liver disease, thyroid disease, thalassemia, anemia, chronic renal disease, and other chronic disease.

### Clinical outcomes.

The clinical outcomes of bacterial and nonbacterial infections are shown in Table 5. Patients with bacterial infection had greater odds of more severe outcomes, including longer hospital stays (OR: 3.67, 95% CI: 2.88–4.69) and intubation/mechanical ventilation (OR: 5.15, 95% CI: 1.90–13.97); they also had lower odds of recovery or improvement of their condition at the time of discharge (OR: 0.11, 95% CI: 0.05–0.23). When adjusting for age and comorbidities, bacterial infection was independently associated with longer hospital stays (aOR: 2.75, 95% CI: 2.08–3.64) and lower odds of recovery or improvement (aOR: 0.14, 95% CI: 0.07–0.31).

**Table 5:** Associations between clinical outcomes and bacterial vs. nonbacterial infections, Nakhon Phanom and Tak provinces, Thailand, April 2017–May 2020

Characteristics	Bacterial Infections (*N* = 528)	Non-Bacterial Infections (*N* = 735)	OR (95% CI)	aOR (95% CI)*
	*n* (%)	*n* (%)		
No. of Days Hospitalized				
0–1	37 (7.0)	48 (6.5)	–	–
2–4	223 (42.2)	526 (71.6)	–	–
≥5	268 (50.8)	161 (21.9)	**3.67 (2.88–4.69)** ^†^	**2.75 (2.08–3.64)** ^†^
Intubation/Mechanical Ventilation	18 (3.4)	5 (0.7)	**5.15 (1.90–13.97)**	2.49 (0.82–7.56)
Discharge Status				
Recovery/Improved	474 (89.9)	724 (98.8)	**0.11 (0.05–0.23)** ^†^	**0.14 (0.07–0.31)** ^†^
Not Improved	49 (9.3)	9 (1.2)	–	–
Deceased	4 (0.8)	0 (0.00)	–	–

aOR = adjusted odds ratio; OR = odds ratio.

* Adjusted for age and existing comorbidities.

^†^ Compared with other categories combined.

### Sensitivity analyses.

Multivariable results did not appreciably differ when stratifying by province (Supplemental Table 5). Most associations remained the same as in combined analyses, but there were several differences. In Nakhon Phanom, patients aged 2–17 years had lower odds of bacterial infection compared with those aged 18–49 years (aOR: 0.43, 95% CI: 0.22–0.84). Use of antibiotics within 72 hours before hospitalization did not differ for patients with bacterial and nonbacterial infections. In Tak, a history of visiting the forest within the previous 2 weeks was independently associated with greater odds of bacterial infection (aOR: 1.89, 95% CI: 1.16–3.07), whereas the presence of jaundice, nausea and/or vomiting, and rash did not significantly differ for patients with bacterial and nonbacterial infections. Furthermore, contact with farm animals was not significantly associated with bacterial infection in either province alone.

## DISCUSSION

This analysis identified several demographic characteristics, exposures, and clinical indicators that were associated with bacterial and nonbacterial etiologies of AUFI. Our results highlight the importance of considering contextual factors to aid in the diagnosis of AUFI, especially in settings with limited resources and laboratory capacities.

Several demographic characteristics and exposure were found to be associated with bacterial infection, indicating that it may be useful to consider patient characteristics when assessing individuals with AUFI. Notably, we found that older age and the presence of comorbidities were both associated with increased odds of bacterial infection, which is consistent with current knowledge on susceptibility to bacterial infections.^36^^–^^38^ Both dengue and *E. coli* may have influenced these findings because our independent analyses showed that patients age ≥50 years and those with comorbidities had greater odds of *E. coli* infection and lower odds of dengue virus infection. In contrast, those with higher hematocrit level and lower white blood cell and platelet counts had a greater odds of dengue infection, consistent with previous literature.^23^^,^^33^^,^^34^ Older patients and those who had comorbidities may have an increased susceptibility to bacterial infection, which can inform diagnostics and treatment decisions. When we adjusted for age and comorbidities, patients with bacterial infections tended to have more severe outcomes including longer hospitalization and decreased odds of recovery at the time of hospital discharge. This is consistent with findings in published literature, in which bacterial infections are more likely than nonbacterial infections to result in sepsis and other complications.^4^^,^^39^ However, this analysis did not consider hematological indicators or complications that arose during hospitalization, so reasons for the severity of bacterial infections were not assessed. Proper diagnosis of bacterial pathogens and early appropriate antibiotic treatment may improve patient outcomes.

Our findings also show that individuals who took antibiotics in the 72 hours before hospital presentation had greater odds of bacterial infection. Differing symptoms and symptom severity between bacterial and nonbacterial infections may have influenced the likelihood of taking antibiotics before hospital presentation, but further research should be done to assess drivers of antibiotic use. The high proportion (10%) of participants with nonbacterial infections who took antibiotics before seeking care should not be overlooked. Providers can encourage patients to seek proper clinical and/or laboratory diagnosis before beginning antimicrobial treatment to reduce the chances of not being able to confirm bacterial infections and also because of the substantial burden of AMR in Thailand and the Southeast Asia region.^9^^,^^40^^,^^41^

Several signs and symptoms were associated with bacterial versus nonbacterial infections. Patients presenting with jaundice had greater odds of bacterial infection, which may be reflective of current knowledge that several bacterial infections, including leptospirosis and rickettsiosis, can cause jaundice, especially when the infection is severe or has resulted in sepsis.^17^^,^^39^^,^^42^ However, diagnostic tests for viral hepatitis were not included in the analysis, and results may have differed if hepatitis was included as a nonbacterial infection in this analysis. We also found that chest pain and pallor were associated with bacterial infections. Although some nonbacterial pathogens can cause chest pain and pallor, our results indicate that bacterial causes may be more likely among febrile patients in Thailand. Considered together with other symptoms, exposure history, and patient characteristics, the presence of signs and symptoms such as jaundice, chest pain, and pallor may prompt healthcare workers to investigate a potential bacterial etiology, possibly leading to quicker diagnosis and treatment.

Although many infectious etiologies can cause nausea and/or vomiting, muscle pain, and rash, we found these symptoms to be associated with nonbacterial infection. The high prevalence of dengue infections in our analysis, as well as the chikungunya outbreak identified in year 3 of the study, are likely drivers of these findings, given that all three signs and symptoms are known to be characteristic of dengue and/or chikungunya infections.^43^^,^^44^ Because of the high prevalence of dengue and chikungunya in Thailand’s border regions,^45^ the presence of nausea and/or vomiting, muscle pain, or rash alongside fever can prompt healthcare professionals to test for both pathogens before prescribing antibiotics.

### Differences by province.

A significantly greater proportion of nonbacterial infections were found in participants from Tak than in those from Nakhon Phanom, which is likely due to its location bordering Myanmar. In Thailand, higher incidences of dengue and malaria are seen in provinces bordering Myanmar than in provinces bordering Laos,^24^^,^^45^ and this was reflected in our findings of higher proportions of both dengue and malaria detections in participants from Tak than in those from Nakhon Phanom. Chikungunya was only identified during year 3 of the study, which corresponds with nationwide dengue and chikungunya outbreaks that were observed in 2019,^43^^,^^46^^,^^47^ and may explain why patients enrolled in the third year of the study were significantly less likely to have bacterial infections.

Although there were not many differences between the combined and individual analyses for Nakhon Phanom and Tak, the provinces are different in terms of populations, geography, common types of illnesses. For instance, melioidosis, *Rickettsia*, *K. pneumoniae*, and *S. agalactiae* were predominantly found in Nakhon Phanom; scrub typhus, chikungunya virus, and malaria were predominantly found in Tak. These differences should be considered in conjunction with clinical presentations and epidemiological information, in diagnostic and treatment decisions at the local level.

### Limitations.

This analysis is subject to at least four limitations. First, only patients admitted to 12 government hospitals were included in the surveillance activity, and only 51.3% of eligible patients consented to participate; therefore, the results may not be representative of the general population or other geographic areas of Thailand. Patients who choose to visit private hospitals or who only have ease of access to smaller clinics or outpatient departments may have different demographic characteristics or behaviors than those included in our analysis. Second, although testing was conducted for an array of pathogens, it was not done for all possible infectious etiologies. A substantial proportion of eligible patients did not have pathogens detected and were excluded from this analysis, but it is possible that some pathogens were not included in the diagnostic tests that were conducted. Our results may have differed if additional pathogens were included. Third, although we conducted pathogen-specific analyses for the most common pathogens detected in this study, the sample size was not large enough to investigate either the associations between indicators and other pathogens of interest or the effects of multiple bacterial or nonbacterial infections in individuals. Future studies with larger sample sizes would be useful for the comprehensive assessment of the etiologies of AUFI. Finally, there is substantial overlap in the clinical presentations and risk factors for pathogens causing AUFI. Our findings are not meant to serve as diagnostic criteria for bacterial or nonbacterial etiologies, but they may be used to inform targeted laboratory testing (e.g., diagnostic testing focusing on bacterial versus testing focusing on viral or parasitic diseases) and treatment options (e.g., an initial treatment regimen centered on use of empiric antibiotics versus one centered on supportive care that might include empiric use of antiviral or antiparasitic agents) based on the available evidence in febrile persons admitted to hospitals.

## CONCLUSION

Accurate diagnosis of the etiologies of AUFI is challenging in Thailand. Our findings may support inferential decision-making for laboratory testing and treatment options for patients who are suspected to have a fever of infectious origin, influencing timely and appropriate treatment. Comprehensive assessment of exposure history, symptoms, and risk factors can aid healthcare professionals in resource-limited settings to narrow down the likely cause of illness. Improvements in the ability to differentiate between bacterial and nonbacterial etiologies of AUFI may help direct clinical and laboratory assessment of patients with AUFI, which could hasten provision of appropriate care and treatment, including appropriate antibiotic use and timely implementation of precautions to reduce onward transmission of infections, thereby reducing AMR and improving the health outcomes of patients with AUFI in Thailand.

## Supplemental Materials

10.4269/ajtmh.23-0731 Supplemental Materials
